# Prediction of land use for the next 30 years using the PLUS model's multi-scenario simulation in Guizhou Province, China

**DOI:** 10.1038/s41598-024-64014-7

**Published:** 2024-06-07

**Authors:** Juncong Liu, Bangyu Liu, Linjing Wu, Haiying Miao, Jiegang Liu, Ke Jiang, Hu Ding, Weichang Gao, Taoze Liu

**Affiliations:** 1 College of Eco-Environment Engineering, Engineering Research Center of Green and Low-Carbon Technology for Plastic Application, Guizhou Minzu University, Guiyang, 550025 China; 2 College of Architectural Engineering, Research Center of Solid Waste Pollution Control and Recycling, Guizhou Minzu University, Guiyang, 550025 China; 3 Institute of Surface-Earth SystemScience, School of Earth System Science, Tianjin University, Tianjin, 300072 China; 4 Upland Flue-Cured Tobacco Quality & Ecology Key Laboratory of CNTC, Guizhou Academy of Tobacco Science, Guiyang, 550081 China

**Keywords:** Environmental impact, Sustainability

## Abstract

Land use changes significantly impact the structure and functioning of ecosystems. The current research focus lies in how to utilize economic and policy instruments to regulate conflicts among stakeholders effectively. The objective is to facilitate rational planning and sustainable development of land utilization resources. The PLUS model integrates a rule-based mining method for land expansion analysis and a CA model based on multi-type stochastic seeding mechanism, which can be used to mine the driving factors of land expansion and predict the patch-level evolution of land use landscapes. Using the PLUS model, a simulation was conducted to study the future land use distribution in the research area over the next 30 years. Based on land use data from Guizhou Province in 2000, 2010, and 2020, a total of 16 driving factors were selected from three aspects: geographical environment, transportation network, and socio-economic conditions. Four scenarios, namely natural development, urban development, ecological conservation, and farmland rotection, were established. Comparative analysis of the simulated differences among the various scenarios was performed. (1) The overall accuracy of the land use simulation using the PLUS model in the study area was 0.983, with a Kappa coefficient of 0.972 and a FoM coefficient of 0.509. The research accuracy meets the simulation requirements. (2) Through the simulation of four different scenarios, the study investigated the land use changes in Guizhou Province over the next 30 years. Each scenario exhibited distinct impacts on land utilization. Comprehensive comparison of the different simulation results revealed that the farmland protection scenario aligns with the sustainable development goals of the research area. Currently, there is a relative scarcity of research on land use simulation, particularly in model application, for Guizhou Province. This study aims to provide a reference for the rational planning of land resources and high-quality urban construction in Guizhou, promoting the high-quality economic development in tandem with advanced ecological and environmental protection.

## Introduction

Land use and land cover change are considered the most significant environmental impacts of human activity^1^. With rapid population growth and accelerated industrialization and urbanization, land use, as a comprehensive entity sustaining human life and production, has undergone major changes globally. These changes have led to severe scarcity and wastage of land resources, as well as widespread encroachment on agricultural land. Consequently, global issues such as land degradation and deteriorating ecological environments have become increasingly critical. Land use and land cover changes have become pressing issues that need to be addressed^2^. Land Use and Land Cover Change (LULCC), a key indicator of natural and economic processes, is essential for revealing the causes and consequences of land dynamics. Moreover, LULCC is deeply influenced by multiple factors such as politics, environment, institutions, and culture. Detecting and assessing these impacts pose significant academic challenges and have become a focal point of research. Findings from these studies offer decision-support to policymakers, enhancing their land management decision-making capabilities^3–5^. As of the twenty-first century, numerous researchers have conducted in-depth and extensive studies on the changing patterns of land use. For instance, alterations in land use can have significant impacts on the climate, leading to changes in surface energy components and carbon balance ^6^. Research indicates that the primary driving factor behind land use change is the population growth in rural areas, while the economic conditions of farmers and land tenure constitute indirect influencing factors^7^. Currently, research on future changes and predictions of land use spatial patterns mainly involves comprehensive analyses of natural, socio-economic, and policy factors^8–10^. We assess the primary variables affecting model accuracy by analyzing feature importance scores derived from the random forest model and examine the similarities and differences among these variables^11^. There are also spatial analysis models based on deep learning that classify satellite images and Geographic Information System (GIS) data to construct land use prediction models^12,13^. Typically, models used to simulate land use change characteristics are predominantly focused on CA-Markov model^14–16^, FLUS model^17–19^, OS-CA model^20^, EL-CA model^21,22^, ANN-CA model^23^, and CLUE-S model^24^, among others. However, these models often treat cities as independent entities with a sole focus on internal drivers, making them unsuitable for simulating urban cluster expansion. They lack flexible mechanisms to deal with changes in multiple types of land use patches, which constrains their ability to simulate land use changes at a fine scale and limits the application of cellular automata (CA) in practical planning and land policy development^10,25,26^. The Patch-generating Land Use Simulation (PLUS) model retains adaptive inertia and roulette competition mechanisms, and further improves the extraction of spatial transformation rules to respond coupled human-ecological dynamics^27^. By incorporating a top-down approach and taking into account both social and natural factors that jointly influence land use change, PLUS effectively addresses the aforementioned issues. Moreover, it offers advantages such as high simulation accuracy, rapid data processing, and the ability to effectively simulate complex evolutions involving multiple land use types. It proves to be a valuable tool for studying the general overview and research data of complex processes in various study areas^10,25,28^. Therefore, this study adopts the PLUC model, which offers higher simulation accuracy, faster data processing, and better adaptability, as the tool and method for system analysis.

The complexity and dynamism of urban expansion render land use change a multifactorial and intricate dynamic process^29^. Previous studies have analyzed historical land use or used a combination of Volunteered Geographic Information and satellite remote sensing data to generate land use data, but these approaches do not fully understand the mechanisms of land type evolution and lack consideration of multiple objectives under different development orientations^30,31^. Additionally, these studies lack consideration for multiple objectives under various development orientations^32^. With the advancement of national regional development strategies such as the "Belt and Road Initiative," the Yangtze River Economic Belt, the Greater Bay Area of Guangdong, Hong Kong, and Macau, and the Chengdu-Chongqing Economic Circle, Guizhou Province is deeply integrated into these initiatives. As the province embraces new development opportunities and progresses steadily towards urbanization, it faces the urgent need to address issues such as significant farmland loss and rapid expansion of construction land to ensure food security and meet the demands of a livable and sustainable ecological environment in the future.

Land use change is a complex and dynamic process influenced by multiple factors. However, research on land use modeling in Guizhou Province is limited, and existing studies based on historical land use analysis may not fully comprehend the mechanisms of land use evolution for different land types. This study focuses on the multi-scenario simulation of land-use evolution, providing additional insights into the current land-use analysis. By considering different development orientations, including natural development, urban prioritization, farmland protection, and ecological civilization construction, the research aims to simulate future changes in land-use structure. Spatio-temporal modeling of patch land development dynamics is crucial for sustaining urban development^33^. Ultimately, this work aims to offer valuable references and support for the rational planning and sustainable development of land-use resources in Guizhou Province.

## Materials and methods

### Study area

**Figure 1:**
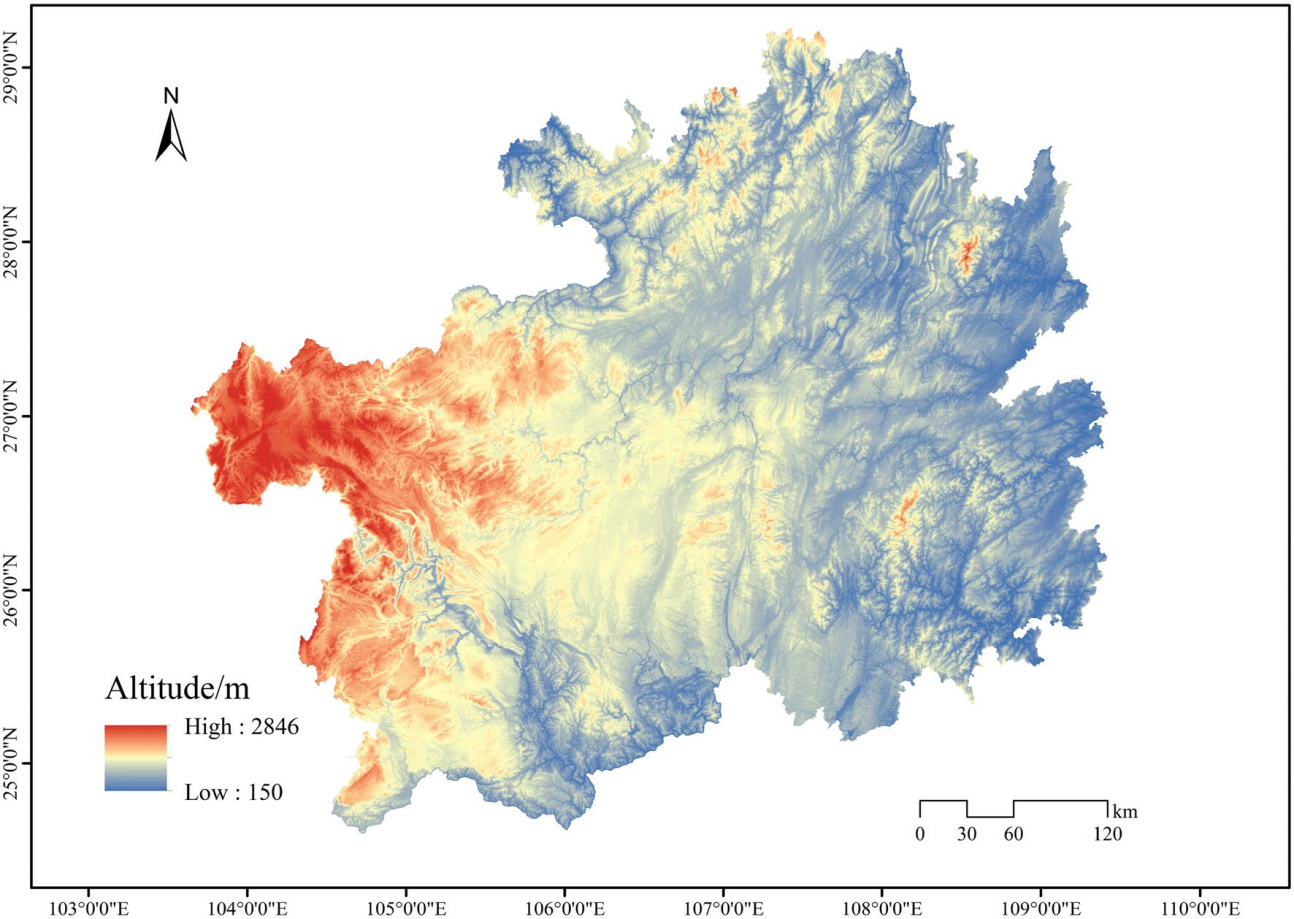
Diagram of the study area.

Guizhou Province is situated in the southwestern inland region of China, between 103° 31′ to 109° 30′ east longitude and 24°30′ to 29°13′ north latitude. The terrain slopes from west to east, tilting towards the north, east, and south sides from the central part. The annual precipitation ranges from 1100 to 1300 mm (Fig. 1). Notably, Guizhou is the only province in China without plains. It features four major mountain ranges: Wumeng Mountains, Dalou Mountains, Miaoling Mountains, and Wuling Mountains. The total land area of the province is 176,200 square kilometers, with mountains and hills accounting for 92.5% of the total area. The karst landscape, covering an area of 109,000 square kilometers, represents 61.9% of the province's total land area. The dominant soil types are yellow soil, red soil, laterite soil, and paddy soil. Limestone and dolomite, forming extensive karst formations, are widespread. Guizhou Province is at the heart of the China Southwest Karst distribution region, one of the world's three major karst concentration areas. As such, it serves as a natural encyclopedia for karst geology.

### Data source and processing

The research dataset includes land-use data for the years 2000, 2010, and 2020 in the study area, sourced from the China Academy of Sciences' Resource and Environment Science Data Center. The data underwent stitching, clipping, and reclassification in ArcMap 10.8.1 to obtain the current land-use status in Guizhou Province. The land-use types are categorized into six classes: cropland, woodland, grassland, water bodies, construction land, and unused land. Subsequently, the data were standardized in ArcMap 10.8.1 to ensure uniform grid rows and columns and consistent projection coordinate systems with other driving factor data. To construct the PLUS model, relevant driving factors were carefully selected based on considerations such as the current state of the study area, data quantifiability, data availability, and correlation. Sixteen influential driving factors were chosen, including Digital Elevation Model (DEM), slope, aspect, annual average temperature, annual precipitation, soil type, population, GDP, distances to various levels of roads (primary, secondary, tertiary, quaternary, and expressways), distances to railways, distances to county government seats, and distances to rivers. See Table 1 for data sources. These factors were divided into natural and social driving factors based on their characteristics. All data were preprocessed using ArcMap 10.8.1, and a consistent resolution of 30 m × 30 m was applied through resampling.

**Table 1:** Data of land use simulation and prediction.

Data type	Data name	Data sources
Natural factors	DEM	Geospatial Data Cloud (https://www.gscloud.cn/)
	Elevation	
	Slope direction	
	Average annual temperature	Resource and Environmental Science and Data Center (https://www.resdc.cn/)
	Land use data	
	Soil type	
	Annual precipitation	China Meteorological Data Service Center (http://data.cma.cn/)
Socio-economic data	Population	Resource and Environmental Science and Data Center (https://www.resdc.cn/)
	GDP	
	Distance to railroad	OpenStreetMap (https://www.openstreetmap.org/)
	Distance to highway	
	Distance to primary roads	
	Distance to secondary roads	
	Distance to tertiary roads	
	Distance to class IV roads	
	Distance to water	
	Distance to County Government	

## Methods

### PLUS model

**Figure 2:**
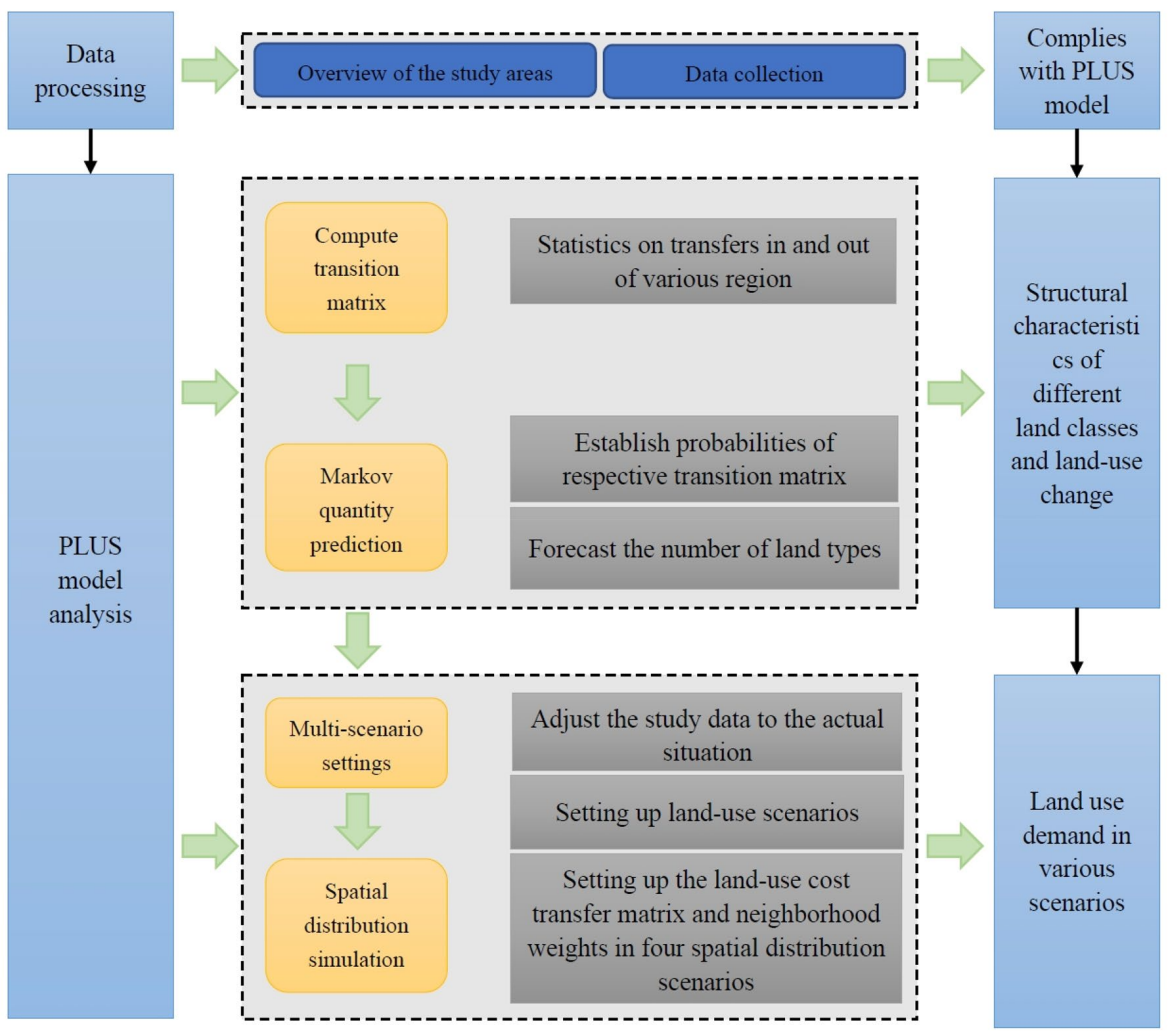
Research implementation processes.

The PLUS model is a land-use simulation model developed by the HMSCIL@CUG Laboratory at China University of Geosciences^10^. The model exhibits flexibility in handling various types of land use patch changes and is suitable for simulating land use changes at the patch scale^9^. The specific steps of this study are illustrated in Fig. 2.

### Transfer matrix and Markov quantity prediction

(1) Transfer matrix

The transition matrix provides a comprehensive and specific depiction of the changes in land use structure and transformation directions between different land use types in the region. This method is rooted in the quantitative description of state transitions and system states in systems analysis. It calculates the transitions into and out of each land use type, thereby reflecting the land use type structure at the beginning and end of the study and revealing the changes in land use type transitions during different study periods^34^. The expression for the transition matrix is as follows:

$${S}_{ij}=\left|\begin{array}{cc}\begin{array}{ccc}{S}_{11}& {S}_{12}& {S}_{13}\end{array}& \begin{array}{ccc}{S}_{14}& \cdots & {S}_{1n}\end{array}\\ \begin{array}{ccc}\begin{array}{c}{S}_{21}\\ {S}_{31}\\ \begin{array}{c}{S}_{41}\\ \cdots \\ {S}_{n1}\end{array}\end{array}& \begin{array}{c}{S}_{22}\\ {S}_{32}\\ \begin{array}{c}{S}_{42}\\ \cdots \\ {S}_{n2}\end{array}\end{array}& \begin{array}{c}{S}_{23}\\ {S}_{33}\\ \begin{array}{c}{S}_{43}\\ \cdots \\ {S}_{n3}\end{array}\end{array}\end{array}& \begin{array}{ccc}\begin{array}{c}{S}_{24}\\ {S}_{34}\\ \begin{array}{c}{S}_{44}\\ \cdots \\ {S}_{n4}\end{array}\end{array}& \begin{array}{c}\cdots \\ \cdots \\ \begin{array}{c}\cdots \\ \cdots \\ \cdots \end{array}\end{array}& \begin{array}{c}{S}_{2n}\\ {S}_{3n}\\ \begin{array}{c}{S}_{4n}\\ \cdots \\ {S}_{nn}\end{array}\end{array}\end{array}\end{array}\right|$$

In the equation, $S$ represents the area; $n$ denotes the number of land use types; and $ij$ respectively represent the land use types at the beginning and end of the study period. In practical applications, the transition matrix is often represented in tabular form for easier visualization and interpretation.

(2) Markov quantity prediction

Based on the historical transition matrix for Guizhou Province from 2010 to 2020, and considering the requirements set for scenarios such as natural development, urban development, ecological conservation, and farmland protection, individual transition matrix probabilities are established. Using the following formula, land quantities for various land use categories are predicted for the years 2030, 2040, and 2050 under the four simulated scenarios of natural development, urban development, ecological conservation, and farmland protection:

$${S}_{t+1}={S}_{t}+{P}_{ij}$$

In the equation: ${P}_{ij}$ represents the transition matrix from land use category $i$ to land use category $j$, while ${S}_{t+1}$​ and $S$​ respectively denote the land use states at time $t+1$ and time $t$.

### Multiple scenario settings

Based on existing research experience and considering the historical land use transition patterns and planning policies in Guizhou Province, four scenarios are set: natural development, urban development, ecological conservation, and farmland protection^35–41^.

1. Natural Development Scenario: Based on the land use change patterns from 2010 to 2020, this scenario follows the principle of "everything remains unchanged," where each land use type is set to evolve according to natural trends. Using a 10-year interval, the PLUS model's Markov Chain is applied to predict the land use demands under the natural growth scenario for the years 2030, 2040, and 2050. Additionally, the natural development scenario serves as the foundation for other scenario simulations^35^.
2. Urban Development Scenario: Considering the "14th Five-Year Plan for the New Urbanization Development in Guizhou Province" and the "Spatial Master Plan of Guizhou Province (2021–2035)," which outline the development targets for construction land based on the land use transitions from 2010 to 2020, we assume a 20% increase in the conversion of cropland, forestland, and grassland to construction land. Using a 10-year interval, we predict the land use situation under the urban development scenario for the years 2030, 2040, and 2050 in the study area^36,37^.
3. Ecological Conservation Scenario: Drawing from the "Ecological Restoration Plan of Guizhou Province (2021–2035)," "14th Five-Year Plan for the Construction of Guizhou Province's National Ecological Civilization Pilot Zone," "14th Five-Year Plan for the Protection and Utilization of Natural Resources in Guizhou Province," "14th Five-Year Plan for Ecological Environment Protection in Guizhou Province," "14th Five-Year Plan for the Protection and Development of Forestry and Grasslands in Guizhou Province," "14th Five-Year Plan for the Protection of Key Watersheds' Aquatic Ecological Environment in Guizhou Province," and "Soil and Water Conservation Plan of Guizhou Province (2016–2030)," we set a 50% reduction in the conversion of forestland and grassland to construction land and a 30% reduction in the conversion of cropland to construction land based on the land use transitions from 2010 to 2020. Using a 10-year interval, we predict the land use situation under the ecological conservation scenario for the years 2030, 2040, and 2050 in the study area^38,39^.
4. Farmland Protection Scenario: Following the priority sequence of "agricultural land first, ecological land second, and urban land last" as emphasized in the "National Land Spatial Planning Outline (2021–2035)" and the "14th Five-Year Plan for Agricultural and Seed Industry Development in Guizhou Province," we set a 20% reduction in the conversion of forestland and grassland to construction land, and a 30% increase in the conversion probability from forestland and grassland to cropland based on the land use transitions from 2010 to 2020. Additionally, we set a 60% reduction in the conversion of cropland to construction land. Using a 10-year interval, we predict the land use situation under the farmland protection scenario for the years 2030, 2040, and 2050 in the study area^40,41^.

### Spatial distribution simulation parameter configuration

To investigate the future 30-year land use changes in Guizhou Province under different development objectives, four scenario-specific spatial distribution cost transfer matrices and neighborhood weights are established based on previous research and the actual land use quantity and spatial distribution in the study area^35–41^. The land use cost transfer matrices only contain binary values, with 0 indicating no conversion is allowed, and 1 indicating permissible conversion. The neighborhood weights represent the expansion intensity of each land use type, assisting in decision-making by considering the neighborhood effects generated by different land use types, and their values range between [0, 1], with larger values indicating stronger neighborhood influences and expansion capabilities. The cost matrices for each scenario (Table 2) and their corresponding weights (Table 3) are as follows:

**Table 2:** Land use conversion cost matrix for each scenario.

	Natural Development Scenario						Urban Development Scenario						Ecological Conservation Scenario						Cropland Protection Scenario					
	a	b	c	d	e	f	a	b	c	d	e	f	a	b	c	d	e	f	a	b	c	d	e	f
a	1	1	0	0	1	1	1	1	0	0	1	1	1	1	1	1	1	1	1	0	0	0	0	0
b	1	1	1	0	0	1	1	1	0	0	1	1	0	1	0	0	0	0	1	1	1	0	0	1
c	1	1	1	1	1	1	1	1	1	1	1	1	0	1	1	1	0	0	1	1	1	1	1	1
d	1	0	1	1	0	1	0	0	0	1	1	0	0	0	0	1	0	0	1	0	0	1	0	0
e	0	0	0	0	1	0	0	0	0	0	1	0	0	0	0	0	1	0	1	0	0	0	1	0
f	1	1	1	1	1	1	1	1	1	1	1	1	1	1	1	1	1	1	1	1	1	1	1	1

a, b, c, d, e, f represent respectively the land use types in Guizhou Province: a for Farmland, b for Forestland, c for Grassland, d for Water Bodies, e for Built-up land, and f for Unused land.

**Table 3:** The table of Weight of Neighborhood.

Scenario Categories	Farmland	Forestland	Grassland	Water Bodies	Built-up land	Unused land
Natural Development Scenario	0.30	0.50	0.40	0.50	0.80	0.01
Urban Development Scenario	0.30	0.40	0.30	0.50	1.00	0.01
Ecological Conservation Scenario	0.30	1.00	0.80	0.60	0.20	0.01
Cropland Protection Scenario	1.00	0.50	0.30	0.60	0.40	0.01

## Results and discussion

### PLUS model accuracy assessment

In this study, the model simulation accuracy is evaluated using the Kappa coefficient and Figure of Merit (FoM). The Kappa coefficient is a statistical measure of agreement, typically ranging between 0 and 1. The interpretation criteria for Kappa values are as follows: 0.0 to 0.20 (slight), 0.21 to 0.40 (fair), 0.41 to 0.60 (moderate), 0.61 to 0.80 (substantial), and 0.81 to 1 (almost perfect). On the other hand, FoM is an index that reflects the consistency at the unit level and similarity at the pattern level. It provides valuable insights into the model's performance in terms of consistency and similarity of land use patterns. By using these two assessment metrics, the study aims to comprehensively evaluate the accuracy and consistency of the model simulations. High Kappa values and significant FoM scores indicate a robust and reliable model performance, reinforcing the credibility of the land use predictions made in this research.

**Figure 3:**
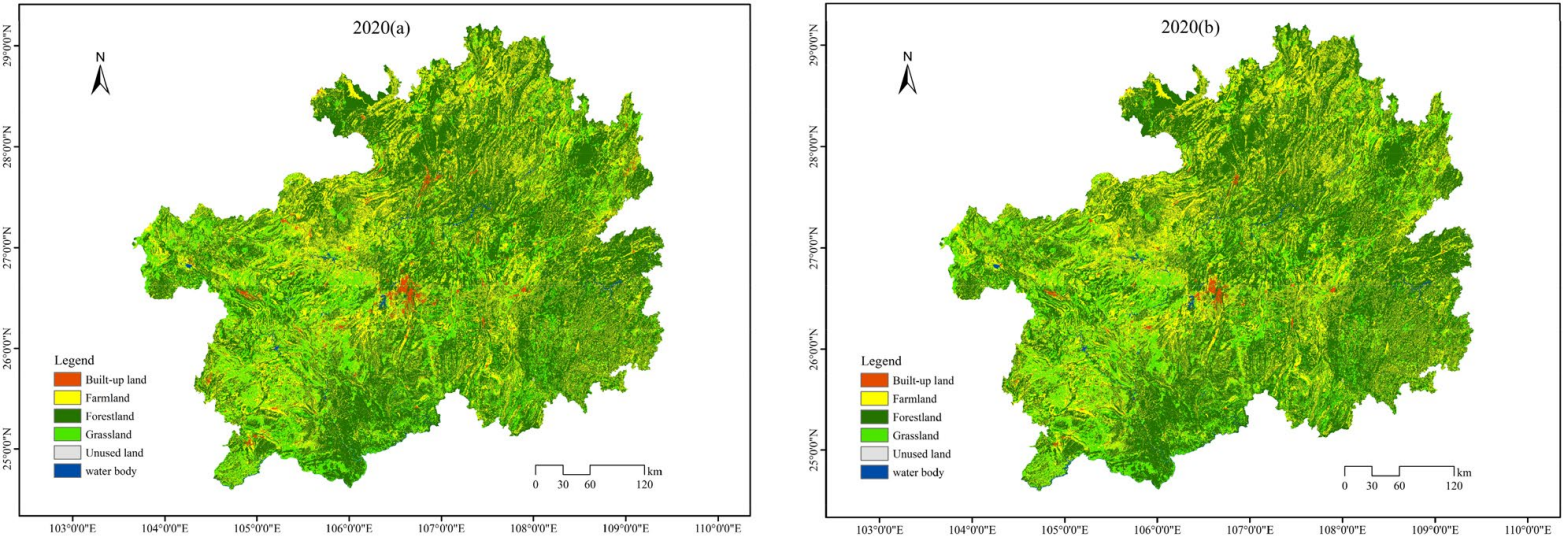
Comparison chart of actual 2020 (**a**) and simulated 2020 (**b**) LUCC.

To verify the simulation accuracy of the constructed PLUS model, this study utilized land use data from 2000 and 2010 to simulate the spatial distribution of land use in 2020. The simulation results were compared with actual conditions to construct a confusion matrix for 2020, which was then used to calculate the overall accuracy and Kappa coefficient of the model. By calculating the overall accuracy, Kappa coefficient, and FoM coefficient, the model's accuracy was verified. As shown in Fig. 3, the spatial distribution obtained by the PLUS model simulation exhibits a high degree of similarity with the actual distribution. The evaluation results indicate that the overall accuracy of the PLUS model simulation in the study area is 0.983, the Kappa coefficient is 0.972, and the FoM coefficient is 0.509. These high values demonstrate that the model performs well and meets the required simulation standards. The PLUS model is suitable for multi-scenario simulations of land use spatial distribution in Guizhou Province.

### Simulated land use scenarios for the years 2030, 2040, and 2050 in Guizhou Province

#### Natural development scenarios

**Figure 4:**
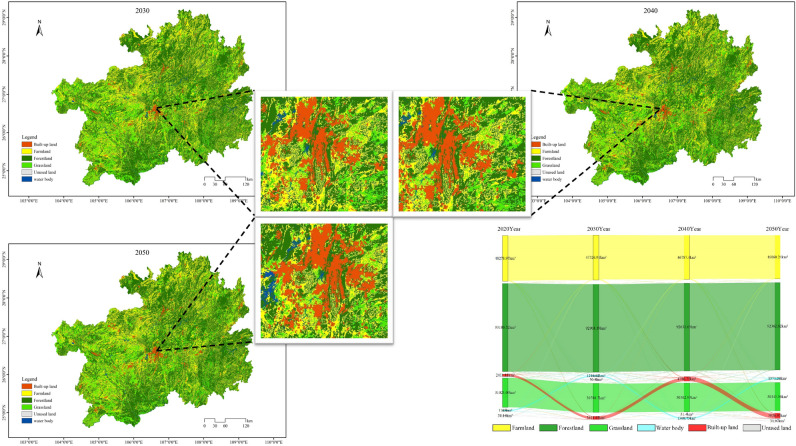
In the context of natural progression, simulations of land utilization for the years 2030, 2040, and 2050, as well as the Sankey diagram, are envisioned.

Based on the analysis of land utilization simulations and the Sankey diagram (Fig. 4) under the scenario of natural progression for the years 2030, 2040, and 2050, it is apparent that as of 2020, forest and grassland collectively accounted for 70.52% of the entire provincial land coverage. However, over the three time points considered in the natural progression scenario, the land area dedicated to cultivated land, forest, and grassland gradually decreased. Besides transfers between these land categories, a significant portion of land was redirected towards urban development. At intervals of 10 years, the area of urban development increased from 2411.12 km^2^ in 2020 to 3611.65 km^2^ in 2030, further expanding to 4786.75 km^2^ in 2040, and reaching 5936.93 km^2^ in 2050. This category displayed the most conspicuous increase in land area and experienced a remarkable growth rate of 146.23% from 2020 to 2050 within the natural progression scenario. The expansion of urban development predominantly radiated from central cities to surrounding regions, with significant changes concentrated in Guizhou City, Zunyi City, and Qiandongnan Prefecture. This phenomenon is closely associated with the accelerated urbanization process and population growth in the study area. It is imperative to note that the expansion of urban development may have irreversible impacts on the natural environment and ecosystems. Therefore, enhancing the planning and management of urban development is of utmost importance. Meanwhile, water bodies and unused land experienced gradual and relatively stable growth among the four land categories. The increase in these areas mainly resulted from the conversion of forest and grassland. This indicates that existing policies concerning water resource utilization and conservation will have a sustained impact on the next 30 years. However, further efforts are needed to strengthen the utilization of unused land and ensure sustainable land use practices.

#### Urban development scenarios

**Figure 5:**
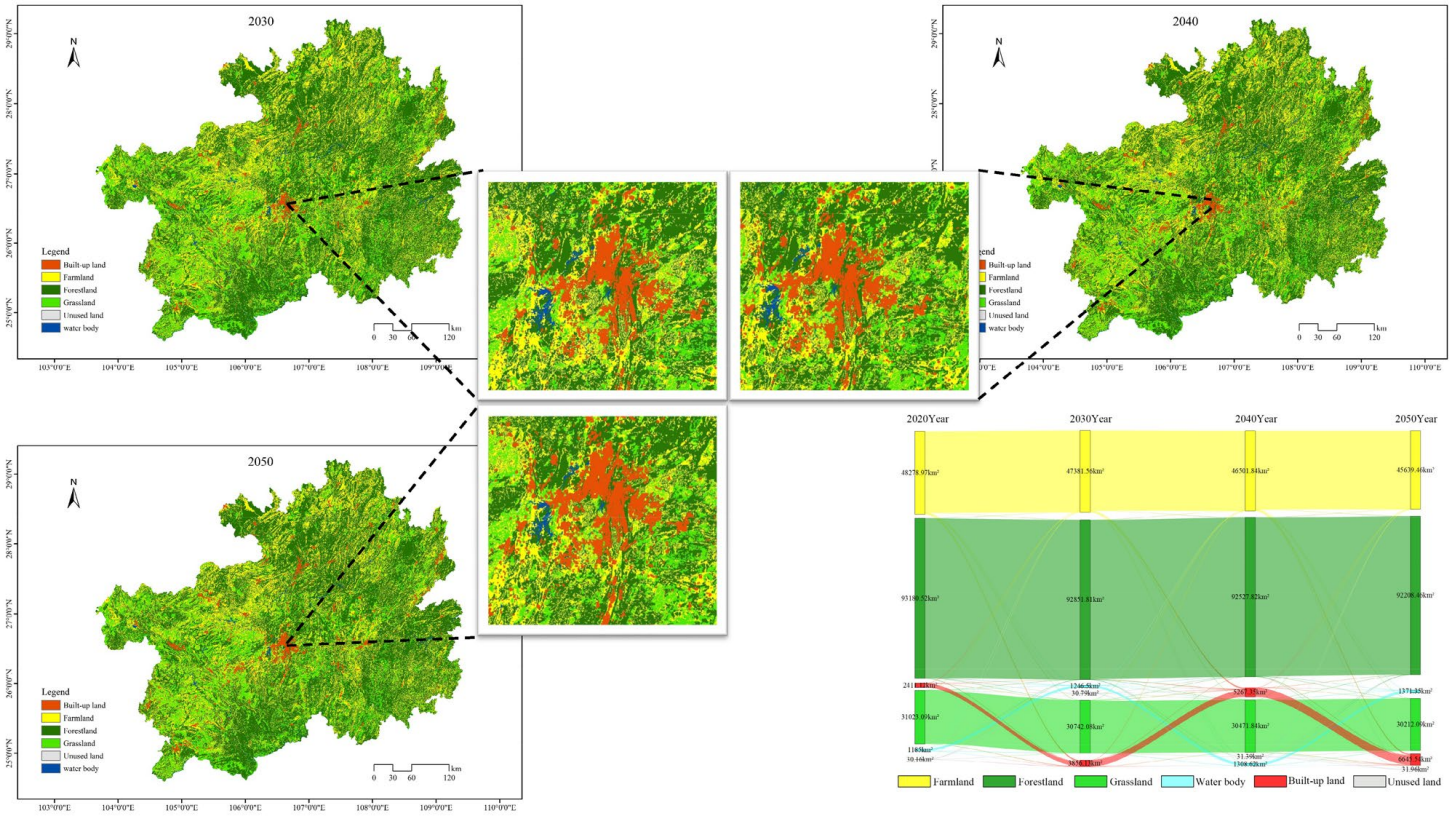
Under the urban development scenario, the land utilization simulations and Sankey diagrams for the years 2030, 2040, and 2050 are depicted.

Through the analysis of Fig. 5, it is evident that under the urban development scenario, the area of constructed land in the study area increased from 2411.12 km^2^ in 2020 to 6645.54 km^2^ in 2050, representing a remarkable growth of 175.62%. The expansion of constructed land predominantly radiated outward from the central cities and prefectures, with significant changes concentrated in Guizhou City, Zunyi City, Bijie City, Anshun City, and Qiandongnan Prefecture. This expansion is closely related to the local economic development status in the study area. The five cities and prefectures have demonstrated strong capabilities to attract population concentration, possess well-developed transportation infrastructure, and feature relatively flat terrain, all contributing to the continuous expansion of constructed land. However, this expansion has led to a substantial reduction in cultivated land, with the cultivated land area decreasing to 45,639.46 km^2^ by 2050, representing a reduction of 2639.51 km^2^ compared to 2020. Additionally, ecological land primarily consisting of forest and grassland will decrease by 1783.06 km^2^. Furthermore, under the urban development scenario, the water area is projected to reach 1371.35 km^2^ in 2050, an increase of 186.35 km^2^ from 2020, mainly sourced from the conversion of forest and grassland. Compared to the natural progression scenario, the urban development scenario in 2050 exhibits a drastic reduction in cultivated land and ecological land area, along with a substantial increase in constructed land. This trend poses a severe threat to the protection of cultivated land and ecological development in the study area.

#### Ecological protection scenarios

**Figure 6:**
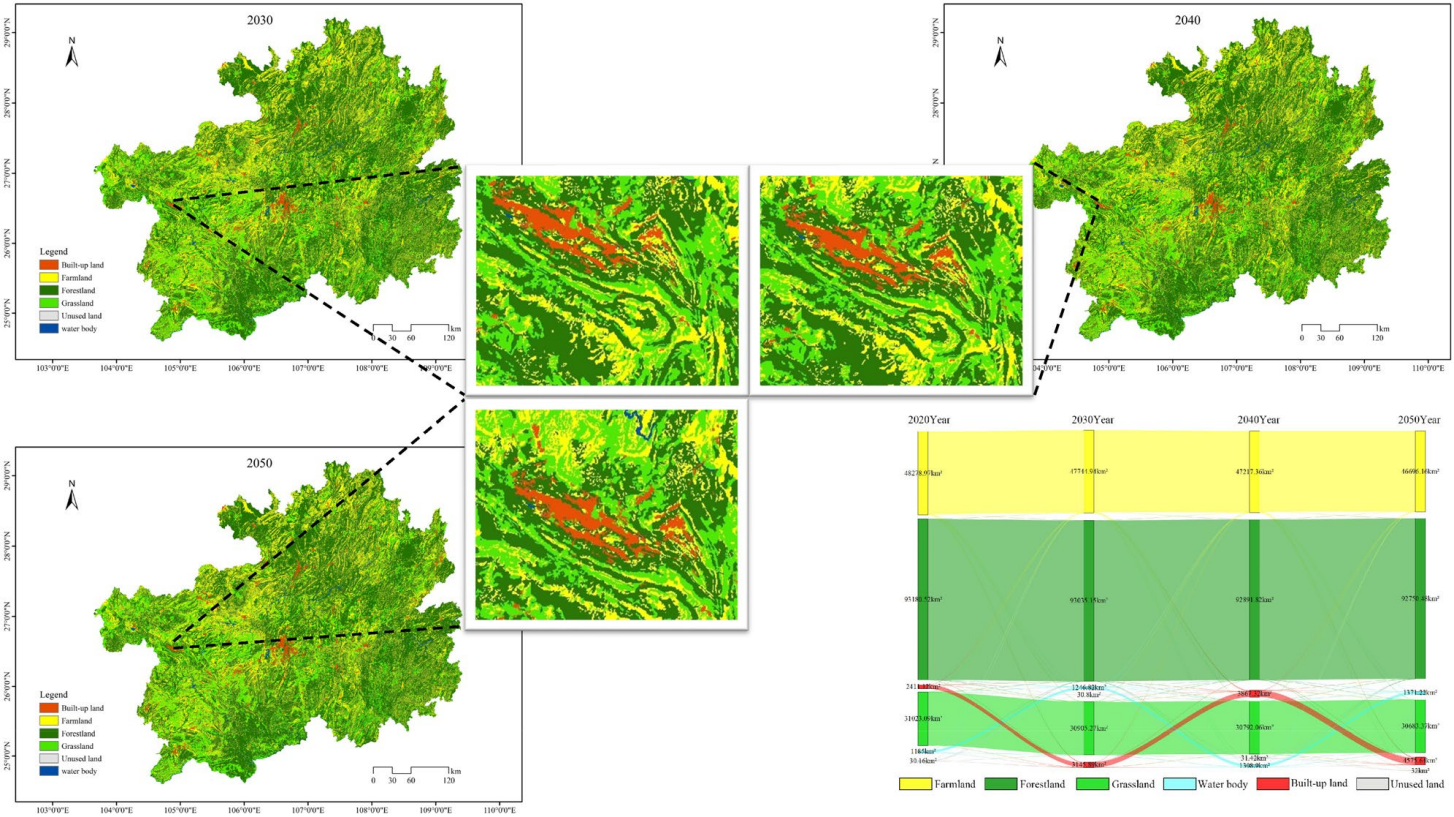
Under the ecological conservation scenario, the simulations of land utilization for the years 2030, 2040, and 2050, along with the corresponding Sankey diagram, are depicted.

Taking into account Fig. 6, under the ecological conservation scenario, the total area of ecological land, including both forest and grassland, in the study area for the year 2050 is 123,433.85 km^2^, marking a reduction of 769.76 km^2^ compared to the year 2020. The decrease in ecological land area under the ecological conservation scenario is slightly higher than that observed in the natural progression, urban development, and cultivated land protection scenarios, with increments of 725.47 km^2^, 1,013.30 km^2^, and 453.26 km^2^, respectively. This enhancement is primarily attributed to the improvement in the situation of constructed land encroachment on ecological land, leading to more effective control over the reduction of ecological land area. The regions experiencing growth in ecological land area are primarily situated around national-level nature reserves, such as Fodengshan, Fanjingshan, Leigongshan, and the Central Subtropical Evergreen Broadleaved Forest, as well as in the vicinity of Qiannan, Qiandongnan, Qianxinan, Liupanshui, and Bijie. Compared to the urban development scenario, the ecological conservation scenario exhibits a slower rate of cultivated land reduction and a minor decrease in constructed land area. While this scenario effectively alleviates the encroachment of infrastructure and public service facilities on ecological land and somewhat limits the expansion of constructed land, its efforts in safeguarding cultivated land remain limited. As a result, this situation may pose challenges to ensuring food security in Guizhou Province.

#### Cultivated land conservation scenario

**Figure 7:**
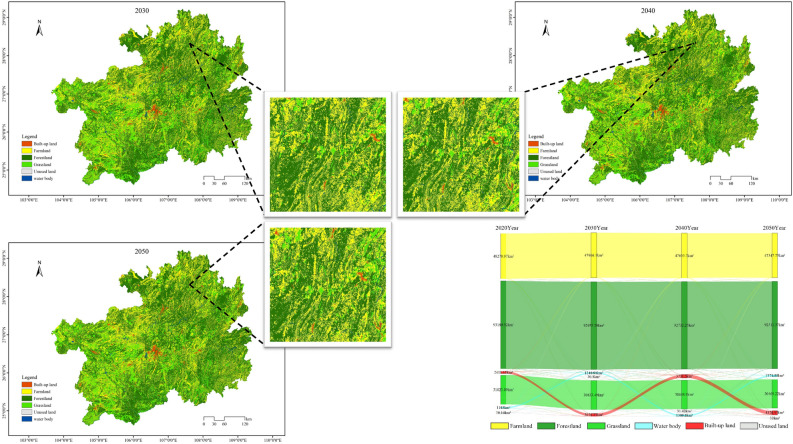
The land utilization simulations and Sankey diagrams for the years 2030, 2040, and 2050 under the Cultivated Land Conservation Scenario.

Under the Cultivated Land Conservation Scenario (Fig. 7), the area of constructed land in 2050 is 4376.97 km^2^, representing the lowest expansion rate among the four simulated scenarios, indicating that the growth trend is effectively controlled. However, compared to the year 2020, the constructed land area has increased by 1965.85 km^2^, showing a continuous expansion trend. In the Cultivated Land Conservation Scenario, the cultivated land area in 2050 reaches 47,347.75 km^2^. Compared to the Natural Progression Scenario, Urban Development Scenario, and Ecological Conservation Scenario, the cultivated land area has increased by 1287.51 km^2^, 1708.29 km^2^, and 651.59 km^2^, respectively, with the main increment concentrated in the central and northern regions of Guizhou Province. Regarding the ecological land area, in 2050, forest and grassland show slight differences compared to the Natural Progression Scenario, Urban Development Scenario, and Ecological Conservation Scenario, with an increase of 272.21 km^2^, 560.04 km^2^, and a decrease of 453.26 km^2^, respectively. The Cultivated Land Conservation Scenario not only ensures the orderly expansion of constructed land, reducing the rate of cultivated land conversion to other land categories, but also promotes efficient protection of cultivated land and guarantees food security. Moreover, it prevents significant loss of forest and grassland, fostering a harmonious development between ecological and economic aspects.

#### Comparative analysis of four scenarios

In the coming 30 years, we ought to direct more attention towards the potential ecological and resource value of arable land, forests, and grasslands, avoiding excessive development and ecological damage. Four scenarios correspond to different developmental directions within the study area. Under the 'Natural Development' scenario, each land-use type evolves according to natural trends, with a gradual reduction in the area of arable land, forests, and grasslands. They shift more towards construction land, except for some interconversion between them. In the 'Urban Development' scenario, the emphasis is on accelerating the development of urban agglomerations and improving urban construction, particularly focusing on infrastructure development. Compared to the 'Ecological Conservation' and 'Arable Land Protection' scenarios, the expansion of construction land is more evident, especially in cities and prefectures like Guiyang, Zunyi, Bijie, Anshun, and Qiandongnan, where construction land shows concentrated growth around surrounding towns. Under the 'Ecological Conservation' scenario, the aim is to consolidate the existing achievements in environmental protection and enhance ecological restoration efforts. This scenario prioritizes the enhancement of management in national-level nature reserves such as Foding Mountain, Fanjing Mountain, Leigong Mountain, and the Mid-subtropical Evergreen Broadleaf Forest. Consequently, the distribution of ecological land in these areas becomes denser, effectively mitigating the phenomenon of substantial encroachment by construction land. In the 'Arable Land Protection' scenario, the objective is to implement the arable land protection system and strengthen food security safeguards. As a result, the distribution of arable land in the central and northern parts of the study area becomes more concentrated, with a significant reduction in the rate of decrease. The loss of ecological land area is minimized, and the expansion of construction land is effectively controlled. Regardless of the scenario, as the urbanization process advances within the study area and socio-economic development progresses steadily, the expansion of construction land is inevitable. However, when comprehensively comparing all scenarios, the 'Arable Land Protection' scenario leads to a more rational land-use structure in Guizhou Province over the next 30 years. It holds significant guiding implications for the reasonable allocation of land reserves and resources across various regions in Guizhou Province.

## Conclusions

Based on the analysis of historical land use, this study clarifies the current situation in the research area. Utilizing the PLUS model and considering the unique characteristics of future urban development in the study area, various factors, including the differential impact of roads at different levels on land use changes, have been comprehensively taken into account. Additionally, the control transformation rates for each scenario were integrated into the modeling process. As a result, the future land use structure in the research area has been objectively and accurately simulated. This research provides essential technical support for the future development of the study area. The conclusions drawn from the study are as follows:

1. By conducting simulations of land use in Guizhou Province for the year 2020 and analyzing the data using the PLUS model, the overall accuracy of the model was found to be 0.983, with a Kappa coefficient of 0.972 and a FoM coefficient of 0.509. The research findings demonstrate that the model exhibits a high level of precision in simulating various scenarios of land use spatial distribution in Guizhou Province, meeting the requirements for accurate simulations. As a result, this model is deemed suitable for conducting land use scenario simulations in Guizhou Province and can be relied upon for future research and decision-making processes.
2. With the advancement of socio-economic development and the urbanization process, the expansion of construction land seems inevitable. However, uncontrolled expansion poses a significant threat to regional ecology and food security. The policies in Guizhou Province reflect the ongoing contradiction between development and protection. As we look towards the future, it is crucial to address the interconnections between different planning policies and strike a balance between development and conservation. Simultaneously, we must prudently manage the growth of construction land in the vicinity of central cities and towns, while improving the efficiency of infrastructure land utilization through spatial intensification.
3. From the results of multiple scenario simulations, the regions experiencing significant land use changes under the "Natural Development" scenario are primarily concentrated in areas such as Guiyang City, Zunyi City, and Qiandongnan Prefecture. Over the next 30 years, there is a gradual decrease in the area of arable land, forests, and grasslands, with more land being converted into construction land. Therefore, it is crucial to pay greater attention to the potential ecological and resource value of arable land, forests, and grasslands, and to avoid excessive development and ecological degradation. Under the "Urban Development" scenario, construction land experiences the highest spatial concentration around central urban areas, but ecological land and arable land face significant losses compared to the other three scenarios. In the "Ecological Conservation" scenario, ecological land exhibits the densest spatial distribution, but the protection of arable land is relatively limited compared to the "Arable Land Protection" scenario. By 2050, the "Arable Land Protection" scenario not only safeguards arable land but also effectively controls the encroachment on ecological land. Compared to the "Ecological Conservation" scenario, the difference in ecological land area is only 453.26 km^2^. Additionally, the expansion of construction land is notably smaller in this scenario, demanding a higher level of efficiency and intensity in construction land utilization. In comprehensive comparison, the "Arable Land Protection" scenario emerges as the optimal choice for the future social development of Guizhou Province. It can serve as a reference for decision-makers in Guizhou Province to plan the sustainable development pattern of land use in the future.

### Prospects

Despite the efforts made in this study to enhance the model's predictive accuracy and applicability through multi-scenario settings and a Markov Chain-based prediction method, several limitations remain that warrant improvement and refinement in future research.

Firstly, this study utilized land-use data from 2010 and 2020 to predict land-use changes for 2030, 2040, and 2050. Future research could incorporate data spanning a longer time frame and with higher resolution to better capture long-term trends and short-term fluctuations, thereby enhancing the model's predictive accuracy. Secondly, although multiple policy and planning factors were considered in the model, the specific implementation effects and future changes of these factors remain uncertain. Therefore, future research should strengthen interdisciplinary collaboration, integrating Geographic Information Systems (GIS), remote sensing technology, and socio-economic data to construct more comprehensive and dynamic land-use prediction models.Lastly, future research needs to make greater strides in data acquisition, methodological optimization, and interdisciplinary integration to further improve the accuracy and reliability of land-use change prediction models.

## Data Availability

The study's original contributions are detailed in the article/supplementary material; any further questions should be directed to the corresponding author.
